# Reactivity studies of pincer bis-protic N-heterocyclic carbene complexes of platinum and palladium under basic conditions

**DOI:** 10.3762/bjoc.12.126

**Published:** 2016-06-28

**Authors:** David C Marelius, Curtis E Moore, Arnold L Rheingold, Douglas B Grotjahn

**Affiliations:** 1 Department of Chemistry and Biochemistry, San Diego State University, San Diego, CA 92182-1030, USA; 2 Department of Chemistry and Biochemistry, University of California San Diego, La Jolla, CA 92093-0358, USA

**Keywords:** NHC, ^15^N NMR spectroscopy, palladium, platinum, protic N-heterocyclic carbene

## Abstract

Bis-protic N-heterocyclic carbene complexes of platinum and palladium (**4**) yield dimeric structures **6** when treated with sodium *tert*-butoxide in CH_2_Cl_2_. The use of a more polar solvent (THF) and a strong base (LiN(iPr)_2_) gave the lithium chloride adducts monobasic complex **7** or analogous dibasic complex **8**.

## Introduction

N-Heterocyclic carbenes (NHCs) have been extensively researched for a number of purposes since 1991 when Arduengo first isolated free NHCs [1–3]. NHCs as ligands have been known even longer. In 1968, Wanzlick and Öfele separately synthesized mercury(II) and chromium(0) imidazol-2-ylidene complexes [3]. Nearly 50 years of NHC ligand research have demonstrated the importance of the electronic and steric effects that can be modified by altering the alkyl or aryl groups on each nitrogen atom. Less common are protic imidazol-2-ylidene (PNHC) ligands with a hydrogen atom on one or both of the stabilizing nitrogens. The synthesis of PNHC complexes has proven to be a challenge, which has limited studies of their reactivity [4–8].

**Figure 1:**
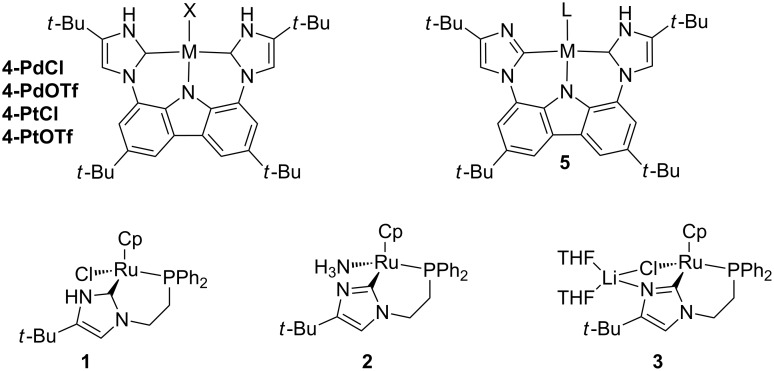
Previously reported PNHC complexes of interest for this work, along with the targeted complex **5**.

Protic imidazol-2-ylidene ligands (e.g., **1**) have been shown to form an imidazolyl ligand (e.g., **2**) after deprotonation with a basic proton-accepting nitrogen (Figure 1). We are unaware of reports on an experimentally determined p*K*_a_ value of a PNHC imidazolidene complex, but looking at related derivatives, Isobe showed that a 2-palladated pyridine was 3.57 p*K*_a_ units more basic than pyridine [9–10]. Considering reactions other than simple proton transfer, imidazol-2-yl complexes have recently been used to bind to a second transition metal [11]. Additionally, Cp*Ir complexes from our group [12] demonstrated heterolysis of the H–H bond of H_2_ and of the C–H bond of acetylene. The same ligand in CpRu complexes **2** and **3** showed heterolysis of dihydrogen [13]. **1** had a much faster ligand exchange rate after ionization as compared to the Cp*Ir analog (ethylene bound in 5 min at rt (CpRu) instead of 16 h at 70 °C (Cp*Ir)). Species **1** could be converted in situ to the hydride and isolated, or generated in situ and used as a transfer hydrogenation catalyst. Interestingly, the ligand substitution rate of ethylene and the heterolysis of dihydrogen was much greater for **3** than for **2**. With only a few papers exploring the utility of these imidazol-2-yl complexes, we aim to extend this to our recently reported pincer bis PNHC complexes **4-PdCl** and **4-PtCl** and their triflato analogs [14]. The design of these complexes was inspired by studies of Kunz et al. on aprotic analogs [15–16].

## Results and Discussion

**Figure 2:**
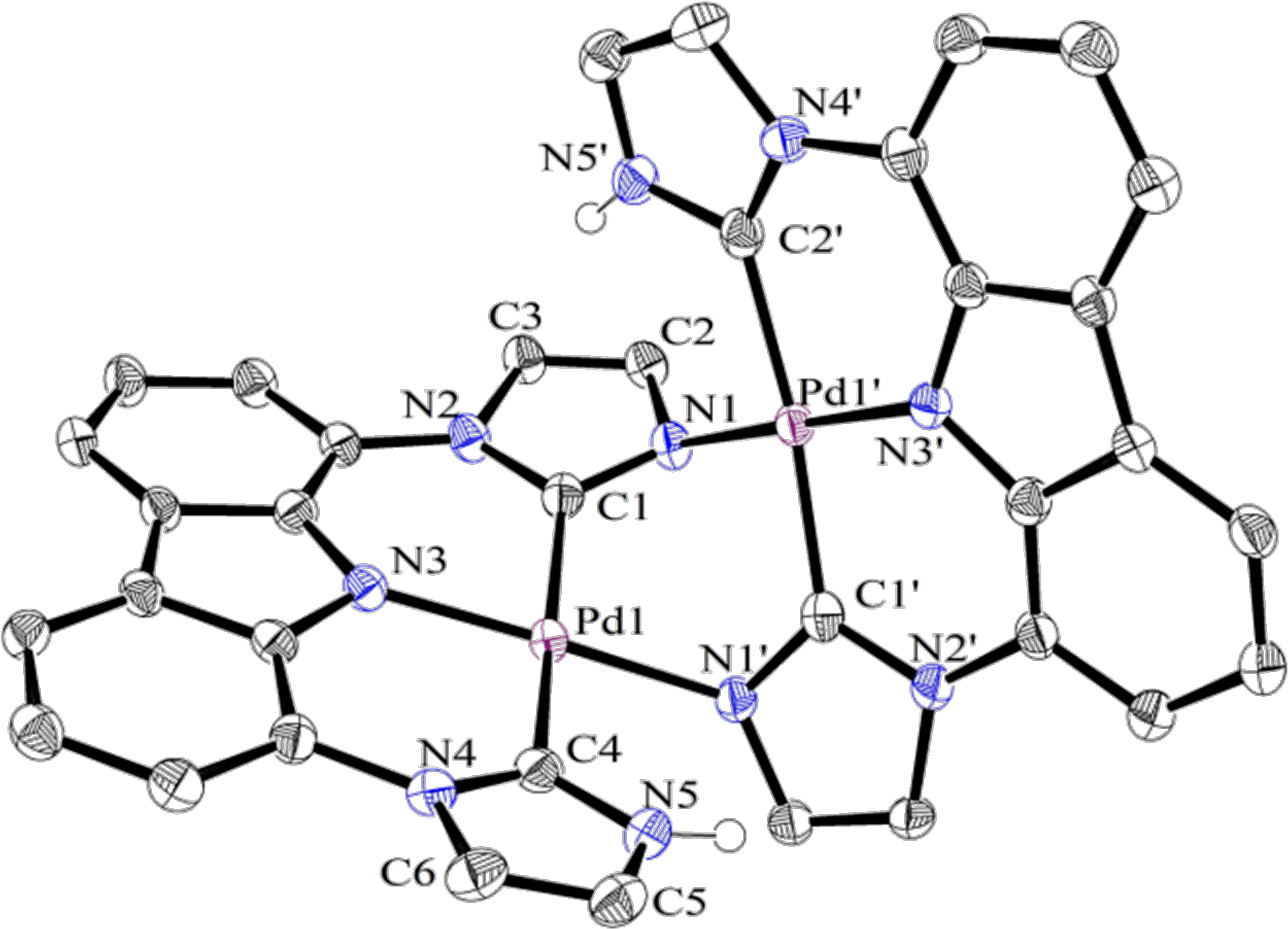
Crystal structure of **6-Pd**. The atoms of the *tert*-butyl groups, C–H bonds, and the solvent have been omitted for clarity.

**Scheme 1:**
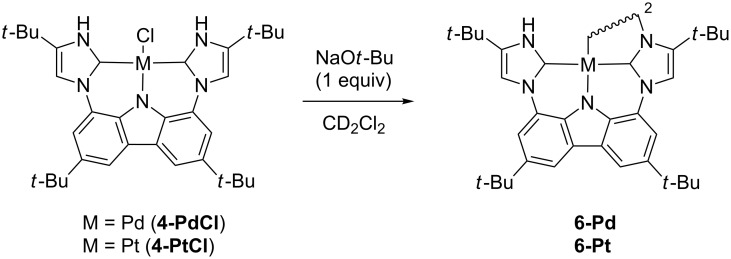
Formation of dimers **6-Pd** and **6-Pt** by addition of NaO*t*-Bu.

The loss of one NH proton from the bis-PNHC complex **4** could lead to structure **5**, a complex concurrently containing a PNHC proton donor and a bond activating imidazol-2-yl unit. In an attempt to form **5**, **4-PdCl** was dissolved in CD_2_Cl_2_, and the solution was saturated with ethylene, followed by the addition of sodium *tert*-butoxide. After 2 h at room temperature, an NMR spectrum was acquired that showed a new, unsymmetrical species, as expected for **5**. Crystals were grown by vapor diffusion of pentanes into benzene and analyzed. Surprisingly, the data showed that the dimer **6-Pd** had formed such that the open site was not filled with ethylene, but rather was occupied by an imidazolyl nitrogen from a second complex (Figure 2). The palladium and platinum dimer complexes, **6-Pd** and **6-Pt**, could be formed by addition of sodium *tert*-butoxide to the chloride analogs (Scheme 1), and isolated in 50–56% yields.

The examination of the dimer crystal structure (see Figure 2 for **6-Pd**) shows strain in the Pd1–N1’ (and Pd1’–N1) bond. This is due to the metal that remains in the plane defined by the three coordinating atoms of the tridentate ligand (i.e., C1, N3, and C4). The fourth donor atom from the other has to bend out of the plane with the N1’ imidazole ring because of the adjacent sterics of the *tert*-butyl groups on the imidazole. The strain can be quantified by examining how far the metal is from the N1 (or N1’)-bound imidazole plane (C1–C2–C3–N1–N2 plane and the symmetry-equivalent atoms): for **6-Pd,** 1.241 Å and, for **6-Pt**, 1.094 Å (Table 1). Further evidence is given by the dihedral angles: Pd1–C1–N1–Pd1’ = −40.4(4)° and 36.5(3)° for **6-Pd** and **6-Pt**, respectively, C1–Pd1–N1’–C1’ = 72.4(3)° and 67.3(2)° for **6-Pd** and **6-Pt**, respectively.

**Table 1:** Key bond lengths (Å) and angles (°) of dimers **6** compared to parent compounds **4**.

	**4-PdCl**	**6-Pd**	**4-PtCl**	**6-Pt**

M–N1	–	2.092(3)	–	2.079(3)
M–N3	1.961(3)	1.962(3)	1.9627(19)	1.969(3)
M–C4	2.006(4), 1.998(4)	2.034(4)	2.007(2), 2.014(2)	2.014(3)
M–C1	–	1.984(4)	–	1.996(3)
N3–M–X	178.09(10)	176.3(1)	179.85(6)	175.6(1)
C1–M–C4	175.43(17)	167.9(2)	176.29(9)	168.3(1)
M out plane^a^	–	1.241	–	1.094

^a^The metal-to-plane distance defined by the five corresponding N-coordinated imidazole atoms; this value would be near zero in the absence of strain.

The NMR results are completely consistent with persistence of the dimers in solution. For monomeric species such as **4-PdCl** and **4-PtCl**, the NH proton resonance is typically downfield shifted with a chemical shift of ca. 11 ppm, whereas this signal is strongly shifted upfield to 8.03 (**6-Pd**) or 8.19 ppm (**6-Pt**). The crystal structures for both **6-Pd** and **6-Pt** show that the NH is located above the pi system of one imidazole ring of the other half of the dimer, which would be expected to shield the NH and cause a significant upfield chemical shift. Moreover, a ROESY experiment on **6-Pt** (Figure S6, Supporting Information File 1) confirms that the NH (N5’, Figure 2) has a through-space interaction with the proton on the imidazole ring (C3, Figure 2), a situation that would not be possible for a monomeric structure.

**Scheme 2:**
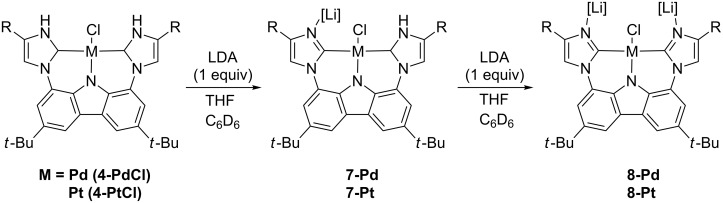
Formation of **7** and **8** by addition of LiN(iPr)_2_ (1 or 2 equiv) (R = *tert*-butyl).

Attempts to synthesize **5** using sodium alkoxide bases led to the formation of dimer structures **6** with presumed loss of NaCl. Therefore, lithium chloride adducts **7** were targeted because LiCl adduct **3** was isolable yet highly reactive. As demonstrated by NMR spectroscopy, the dissolution of **4-PdCl** in a mixture of THF (0.7 mL) and C_6_D_6_ (0.1 mL) followed by the addition of one equivalent of LiN(iPr)_2_ deprotonates one of the PNHC complexes. This gives **7-Pd**, without evidence of dimer formation (Scheme 2). The addition of a second equivalent of LiN(iPr)_2_ deprotonates the second PNHC complex, giving **8-Pd**. The ^1^H NMR spectrum for compound **7-Pt** consists of a single NH peak at 10.90 ppm and six aromatic peaks, which all integrate to one proton. The asymmetry is also observed in the ^13^C NMR spectrum, which consists of 18 peaks between 100 and 170 ppm. As for **8-Pt**, the ^1^H NMR spectrum has no peak where the NH peak typically is located, and in the aromatic region there are three peaks. The ^13^C NMR spectrum thus consists of 9 peaks between 100 and 170 ppm, showing the reappearance of symmetry.

^15^N chemical shift data give structural insight (Table 2), as exemplified by **1**–**3** [10]. The Δ**_x_** (difference in ^15^N shifts for compound **x**) is near zero for a PNHC (**1**), maximum for the imidazolyl conjugate base **2**, and slightly less for an imidazolyl lithium chloride adduct **3**. The δ_N_ for the aprotic nitrogen incapable of acid base chemistry (N2) hardly changes, whereas for the protic (N1), the changes depend on its environment. In the following discussion, **1** and **4-PtCl** are considered the reference starting material complexes because they are both neutral PNHC species with M–Cl moieties. Their ∆_x_ values are named as ∆_ref_. For **1**, ∆_ref_ = 1.5, whereas for **4-PdCl**, ∆_ref_ = −13.1. This difference is likely due to a variety of factors related to the electronics of the nonprotic substituent of the PNHC and/or the ring size of the chelates, which is beyond the scope of this paper. For a given metal center and mono- or bis-NHC framework, we want to diagnose the effects of chemical changes at N1. For this purpose, we introduce the quantity ∆∆ = ∆**_x_** − ∆_ref_ to account for the change in Δ**_x_** that accompanies a chemical change from the reference compound to the new species **x**. Looking at complex **6-Pt**, ∆∆ for the NH nitrogen is only 0.3 ppm and only −4.4 ppm for the NM nitrogen. The ∆∆ values for both nitrogens are relatively unchanged as the carbene character of the ligand is still intact. Interestingly, **6-Pd** shows a similar small ∆∆ value for the NH (2.6) but a greater ∆∆ for the NM of 17.4 ppm. This is possibly a result of the differing ring strain in the Pt case (M out of plane 1.094 Å for **6-Pt** vs 1.241 for **6-Pd**) and/or smaller M–N distance (2.079(3) Å for **6-Pt** vs 2.092(3) Å for **6-Pd**, Table 1). Turning now to **7-Pt** and **8-Pt**, we note that the deprotonated nitrogens of **7-Pt** and **8-Pt** show large values of ∆∆ = 80.5 ppm and 77.8 ppm, respectively. This resembles the results for the CpRu species **2** and **3** (∆∆ = 88.0 ppm and 73.2 ppm, respectively). The values of ∆∆ for the deprotonated nitrogens (that bear a Pt or Pd) in the crystallographically characterized dimers **6-Pt** and **6-Pd** are much smaller (17.4, −4.4). The large ∆∆ values for the deprotonated nitrogens of **7-Pt** and **8-Pt** could be due to either structural formulation as a LiCl adduct or free imidazolyl species. However, the dependence of the stability of **7** and **8** on the presence of chloride (see below) argues strongly for a chloride ligand on the central metal. This leads us to assign structures **7-Pt** and **8-Pt** as LiCl-imidazolyl adducts. All attempts at characterization by crystallography have been unsuccessful due to the very water-sensitive nature of **7** and **8**. Therefore, the environment of the lithium and chloride is unknown but it is assumed to be similar to that of **3**, which is formed under very similar conditions. The ^15^N chemical shift data give structural insight that is unavailable by any other means regarding the absence of solid-state structures or meaningfully diagnostic ^7^Li chemical shifts.

**Table 2:** ^15^N chemical shift values (ppm) of complexes in this paper.^a^

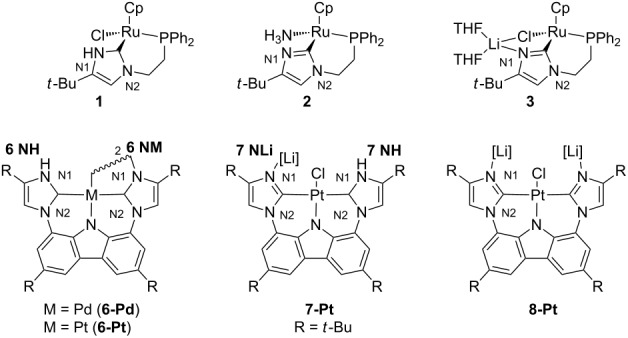				

Complex	N1^b^	N2^c^	∆_x_^d^	∆∆^e^

**1**	−197.1	−198.6	1.5	0 (defined)
**2**	−110.4	−199.9	89.5	88.0
**3**	−122.1	−196.8	74.7	73.2
**4-PtCl**	−211.0	−197.9	−13.1	0 (defined)
**7-Pt NH**	−212.7	−198.4	−14.4	−1.3
**7-Pt Li**	−129.9	−197.3	67.4	80.5
**8-Pt**	−127.4	−192.0	64.7	77.8
**6-Pt NH**	−209.9	−197.0	−12.9	0.3
**6-Pt NPd**	−215.3	−197.8	−17.5	−4.4
**6-Pd NH**	−208.3	−192.6	−15.7	2.6
**6-Pd NPt**	−193.5	−195.8	2.3	17.4

^a^Determined using ^1^H,^15^N gHMBC on natural abundance material in THF (0.7 mL) and benzene-*d*_6_ (0.1 mL) (**4-PtCl**, **7**, **8**), or CD_2_Cl_2_ (**6**). ^b^N1 = either NH or derivative of PNHC. ^c^N2 = aprotic nitrogen incapable of acid base chemistry. ^d^∆**_x_** = N1 − N2. ^e^∆∆ = ∆x − ∆_ref_ where ∆_ref_ = −13.1 for type-**1** compounds and 1.5 for type-**4** compounds.

An indication of the reactivity of imidazolyl complexes was deduced after bubbling H_2_ through a solution of **7-Pt** for 16 h in THF/C_6_D_6_, where only a small amount of **4-PtCl** was regenerated, likely from adventitious water. The addition of AgOTf did not facilitate H_2_ heterolysis, but formed the dimer **6-Pt** (Figure S7, Supporting Information File 1). Bubbling ethylene through a solution of **7-Pt** gave some **4-PtCl**, again likely from adventitious water (Figure S8, Supporting Information File 1). The rigorously dried substrate 1-heptene (20 equiv) was added to **8-Pt**, where no reaction was observed even after heating at 70 °C for 4 h (Figure S9, Supporting Information File 1).

Unlike imidazolyl complex **3**, **7-Pt** showed no tendency to lose LiCl. One possible route to a more labile LiOTf adduct was to deprotonate **4-PtOTf**, but the action of LiN(iPr)_2_ yielded mostly the dimer **6-Pt** (Figure S10, Supporting Information File 1). Alternatively, the deprotonation of [**4-Pt(CH_3_CN)]^+^ OTf^−^** by LiN(iPr)_2_ (1 equiv) gave an analog of **5**, where L = CH_3_CN. However in practice, this typically yielded a number of species (Figure S11, Supporting Information File 1). It appears that the LiCl-imidazolyl adduct complexes **7-Pt** and **8-Pt** have a much slower ligand exchange rate compared to complex **3**. This was expected given the change from ruthenium to platinum [17].

To see if the faster ligand exchange would lead to LiCl loss with palladium, **7-Pd** was synthesized. Unfortunately, similar results were observed with the platinum analog where 1-heptene did not react with **7-Pd** (which was then converted to **8-Pd** by addition of LiN(iPr)_2_). Then AgOTf was added to **8-Pd**, which formed a deprotonated dimer complex. Even with palladium, the loss of the chloride ligand seemed to be too slow.

## Conclusion

In conclusion, attempts at forming an imidazolyl complex from **4-MCl** using sodium alkoxides led to strained dimers **6**. However, **4-MCl** could be deprotonated with either 1 or 2 equiv of LiN(iPr)_2_ to give **7**, an intriguing species with one PNHC ligand and one Li-imidazolyl adduct, or **8**, a bis imidazolyl complex. Unfortunately, substrates could not displace the chloride ligand without formation of dimer **6**, and the deprotonated complexes were water sensitive. The attempts at deprotonating the more labile triflate complex **4-PtOTf** led to the formation of dimer **6-Pt**. To increase the lability of the chloride ligand, species **4-PdCl** and **4-PdOTf** were examined but gave dimer **6-Pd**. In summary, the reactivity of bis-PNHC complexes **4** and bases appears to be dominated by the formation of the dimeric structures. Studies to reduce dimer formation by various means, such as increasing steric hindrance at the imidazolyl nitrogens, will be reported in due course.

## Supporting Information

The Supporting Information contains details on syntheses of **6-Pd** and **6-Pt**, NMR data for **4-PtCl**, **6-Pd**, **6-Pt**, **7-Pt**, and **8-Pt** and Figues S6–S12.

File 1 Experimental information and NMR spectroscopy figures.
