# Supplementary material for: Reactivity studies of pincer bis-protic N-heterocyclic carbene complexes of platinum and palladium under basic conditions
Source: Beilstein J Org Chem. 2016 Jun 28;12:1334–9. doi: 10.3762/bjoc.12.126 (PMC4979752; doi:10.3762/bjoc.12.126)
Supplement: File 1 — Experimental information and NMR spectroscopy figures. [file Beilstein_J_Org_Chem-12-1334-s001.pdf]

Supporting Information
for

Reactivity studies of pincer bis-protic N-heterocyclic carbene complexes of platinum and palladium under basic conditions

David C. Marelius[1], Curtis E. Moore[2], Arnold L. Rheingold[2] and Douglas B. Grotjahn*[1]

Address: [1]Department of Chemistry and Biochemistry, San Diego State University, San Diego, CA 92182-1030, USA and [2]Department of Chemistry and Biochemistry, University of California San Diego, La Jolla, CA 92093-0358, USA

Email: Douglas B. Grotjahn - dbgrotjahn@mail.sdsu.edu

* Corresponding author

Experimental information and NMR spectroscopy figures

TABLE OF CONTENTS

1. NMR data for **6-Pd**, **6-Pt**, **4-PtCl**, **7-Pt**, **8-Pt** S2–S9

2. ROESY 1D spectrum of **6-Pt** S10

3. NMR spectra discussed in paper S11–S16

5. General experimental S17

6. Synthesis of **6-Pd** and **6-Pt** S17–S18

7. Crystal data and structure refinement for **6-Pd** and **6-Pt** S19–S20

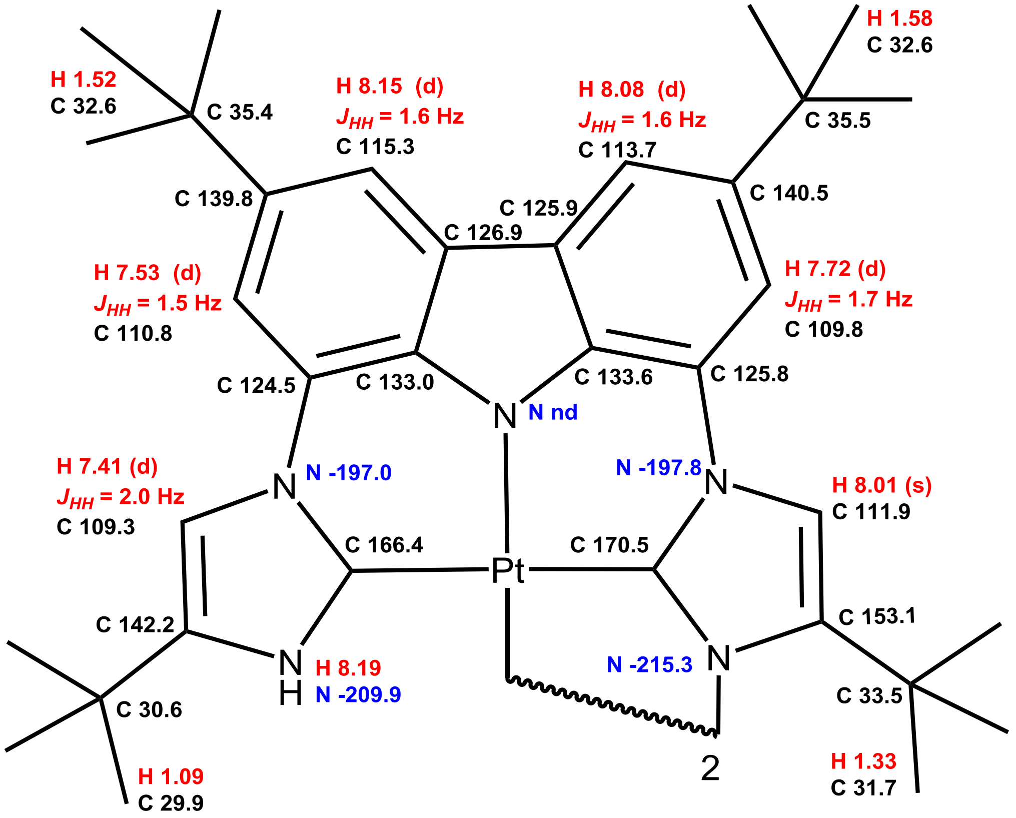

Figure S1: NMR data for **6-Pt** in CD_2Cl_2.

^{1}H - ^{15}N **gHMBCAD**[a]		
H	bonds	N
8.01	3	-215.3
8.01	2	-197.8
7.72	3	-197.8
7.53	3	-197.0
7.41	3	-209.9
7.41	2	-197.0
[a]J_{nxh}= 5 Hz<br>J_{1xh}= 90 Hz		

^{1}H-^{13}C **HSQCAD**	
H	C
8.15	115.3
8.08	113.7
8.01	111.9
7.72	109.8
7.53	110.8
7.41	109.3
1.58	32.6
1.52	32.6
1.33	31.7
1.09	29.9

**gCOSY**
8.19↔7.41
8.15↔7.53
8.08↔7.72

Red = Proton, Black = carbon, Blue = Nitrogen

^{1}H - ^{13}C gHMBC[b]		
H	bonds	C
8.19	2	166.4
8.19	2	142.2
8.19	3	109.3
8.15	3	133.1
8.15	2	126.9(w)
8.15	3	125.9
8.15	4	124.5(w)
8.15	3	110.8
8.15	3	35.4
8.08	3	133.6
8.08	3	126.9
8.08	2	125.9(w)
8.08	3	109.8
8.08	3	35.5
8.01	3	170.5
8.01	2	153.1
8.01	3	125.8(w)
7.72	2	140.5(w)
7.72	3	133.6
7.72	4	125.9
7.72	3	113.7
7.72	3	35.5

^{1}H - ^{13}C gHMBCAD Continued[b]		
H	bonds	C
7.53	2	139.8(w)
7.53	3	133.0
7.53	4	126.9(w)
7.53	2	124.5
7.53	3	115.3
7.53	3	35.4
7.41	3	166.4
7.41	2	142.2
7.41	3	124.5(w)
1.58	3	140.5
1.58	2	35.5
1.58	1,3	32.6
1.52	3	139.8
1.52	2	35.4
1.52	1,3	32.6
1.33	3	153.1
1.33	2	33.5
1.33	1,3	31.7
1.09	3	142.2
1.09	2	30.6
1.09	1,3	29.9
[b] J_{nxh} = 8.0 Hz J_{1xh} = 140.0 Hz		

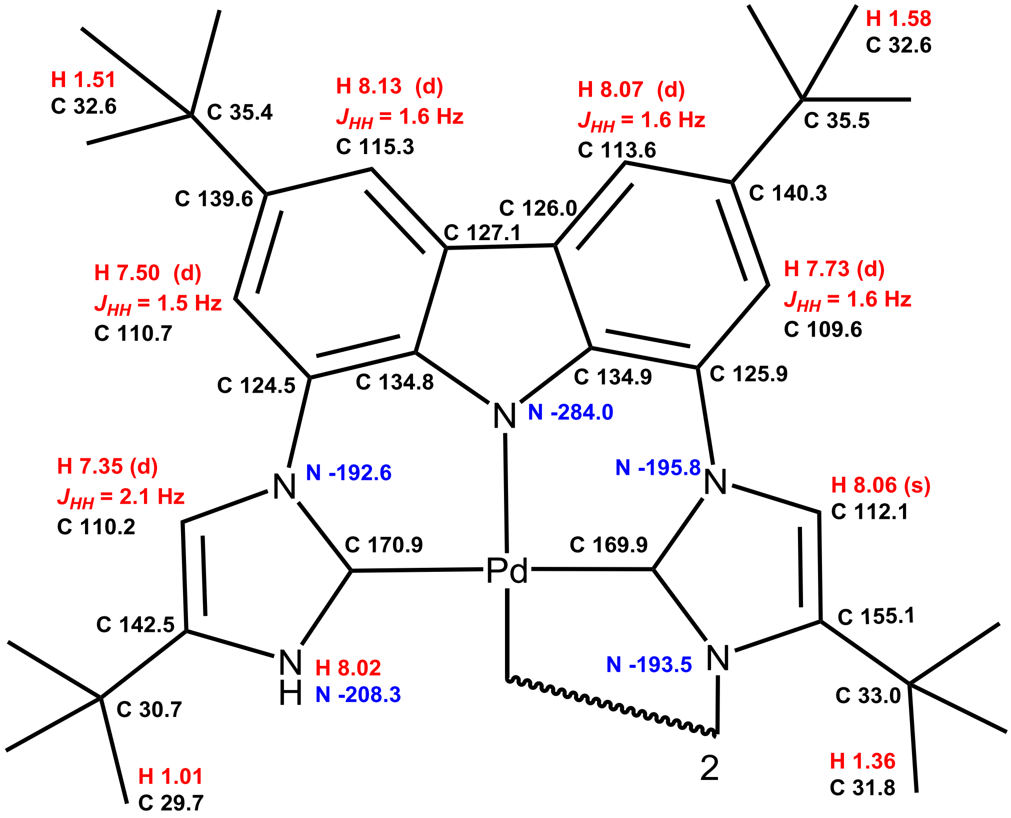

Figure S2: NMR data for **6-Pd** in CD_2Cl_2.

^{1}H - ^{15}N **gHMBCAD**[a]		
H	bonds	N
8.06	2	-195.8
8.06	3	-193.5
7.73	4	-284.0
7.73	3	-195.8
7.50	4	-284.0
7.50	3	-192.6
7.35	3	-208.3
7.35	2	-192.6
[a]J_{nxh}= 5 Hz<br>J_{1xh}= 90 Hz		

^{1}H-^{13}C **HSQCAD**	
H	C
8.13	115.3
8.07	113.6
8.06	112.1
7.73	109.6
7.50	110.7
7.35	110.2
1.58	32.6
1.51	32.6
1.36	31.8
1.01	29.7

**gCOSY**
8.13↔7.50
8.07↔7.73
8.02↔7.35

Red = Proton, Black = carbon, Blue = Nitrogen

^{1}H - ^{13}C gHMBC^{b}		
H	bonds	C
8.13	3	134.8
8.13	3	126.0
8.13	3	110.7
8.13	3	35.4
8.07	3	134.9
8.07	3	127.1
8.07	2	126.0(w)
8.07	3	109.6
8.07	3	35.5
8.06	3	169.9
8.06	2	155.1
8.02	2	170.9(w)
8.02	2	142.5(w)
8.02	3	110.2(w)
7.73	3	134.9
7.73	2	125.9(w)
7.73	3	113.6
7.73	3	35.5
7.50	3	134.8
7.50	2	124.5(w)
7.50	3	115.3
7.50	3	35.4

^{1}H - ^{13}C gHMBCAD Continued^{b}		
H	bonds	C
7.35	3	170.9
7.35	2	142.5
1.58	3	140.3
1.58	2	35.5
1.58	1,3	326
1.51	3	139.6
1.51	2	35.4
1.51	1,3	32.6
1.36	3	155.1
1.36	2	33.0
1.36	1,3	31.8
1.01	3	142.5
1.01	2	30.7
1.01	1,3	29.7
$^{b}J_{nxh}$ = 8.0 Hz J_{1xh} = 146.0 Hz		

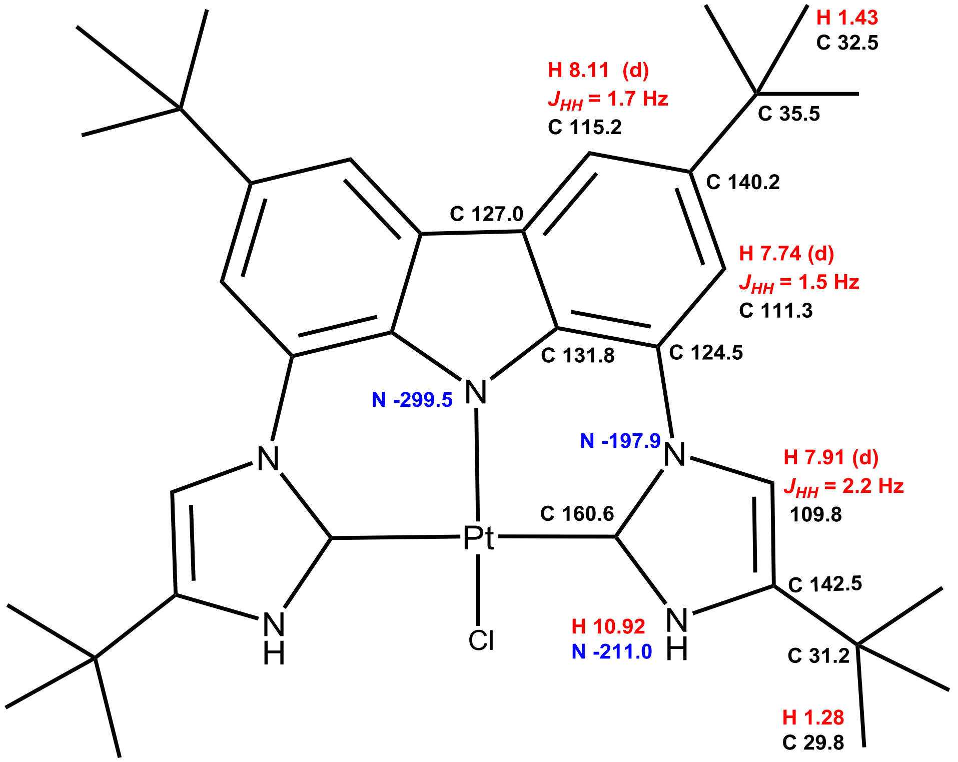

Figure S3: NMR data for **4-PtCl** in THF (0.6 mL) and d_6-benzene (0.1 mL).

^{1}H - ^{13}C gHMBCAD[b]		
H	bonds	C
8.11	3	35.5
8.11	3	111.3
8.11	2	127.0(w)
8.11	3	131.8
7.91	2	142.5
7.91	3	160.6(w)
7.74	3	35.5
7.74	3	115.2
7.74	2	124.5(w)
7.74	3	131.8
1.43	1,3	32.5
1.43	2	35.5
1.43	3	140.2
1.28	1,3	29.8
1.28	2	31.2
1.28	3	142.5
[b]J_{nxh} = 8.0 Hz<br>J_{1xh} = 146.0 Hz		

^{1}H-^{13}C HSQCAD	
H	C
8.11	115.2
7.91	109.8
7.74	111.3
1.43	32.5
1.28	29.8

^{1}H - ^{15}N gHMBCAD[a]		
H	bonds	N
7.91	2	-197.9
7.91	3	-211.0
7.74	3	-197.9
7.74	4	-299.5
[a]J_{nxh}= 5 Hz<br>J_{1xh}= 90 Hz		

gCOSY
10.92↔7.92
8.11↔7.74

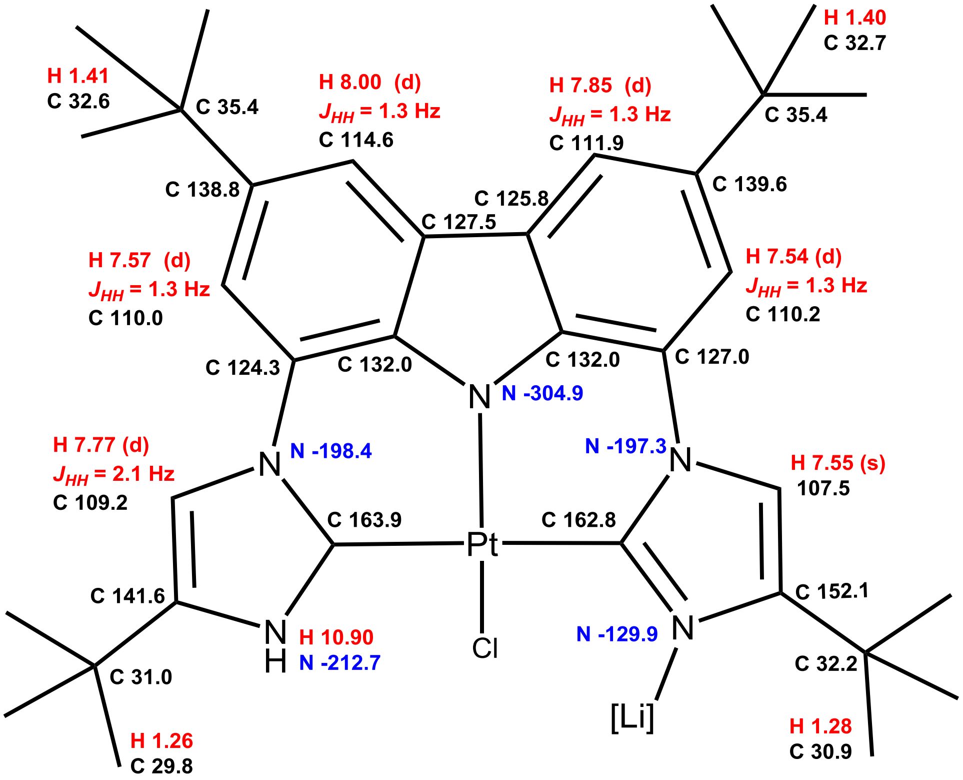

Figure S4: NMR data for **7-Pt** in THF (0.6 mL) and d_6-benzene (0.1 mL).

^{1}H - ^{15}N gHMBCAD[a]		
H	bonds	N
7.77	3	-212.7
7.77	2	-198.4
7.57	4	-304.9
7.57	3	-198.4
7.55	2	-197.3
7.55	3	-129.9
7.54	4	-304.9
7.54	3	-197.3
[a] J_{nxh}= 5 Hz<br>J_{1xh}= 90 Hz		

^{1}H-^{13}C HSQCAD	
H	C
8.00	114.6
7.85	111.9
7.77	109.2
7.57	110.0
7.55	107.5
7.54	110.2
1.41	32.6
1.40	32.7
1.28	30.9
1.26	29.8

^{1}H - ^{15}N gHSQC	
H	N
10.90	-212.7

gCOSY
10.90↔7.77
8.00↔7.57
7.85↔7.54

Red = Proton, Black = carbon, Blue = Nitrogen

^{1}H - ^{13}C gHMBCAD[b]		
H	bonds	C
8.00	3	110.0
8.00	4	124.3(w)
8.00	3	125.8
8.00	2	127.5(w)
8.00	3	132.0
8.00	3	35.4
7.85	3	110.2
7.85	2	125.8(w)
7.85	3	127.5
7.85	3	132.0
7.85	3	35.4
7.77	2	141.6
7.77	3	163.9
7.57	3	114.6
7.57	2	124.3(w)
7.57	4	127.5(w)
7.57	3	132.0
7.57	2	138.8(w)
7.57	3	35.4
7.55	2	152.1
7.55	3	162.8

^{1}H - ^{13}C gHMBCAD Continued[b]		
H	bonds	C
7.54	3	111.9
7.54	4	125.8(w)
7.54	2	127.0
7.54	3	132.0
7.54	2	139.6(w)
7.54	3	35.4
1.41	1,3	32.6
1.41	2	35.4
1.41	3	138.8
1.40	1,3	32.7
1.40	2	35.4
1.40	3	139.6
1.28	1,3	30.9
1.28	2	32.2
1.28	3	152.1
1.26	1,3	29.8
1.26	2	31.0
1.26	3	141.6
[b] J_{nxh} = 8.0 Hz<br>J_{1xh} = 146.0 Hz		

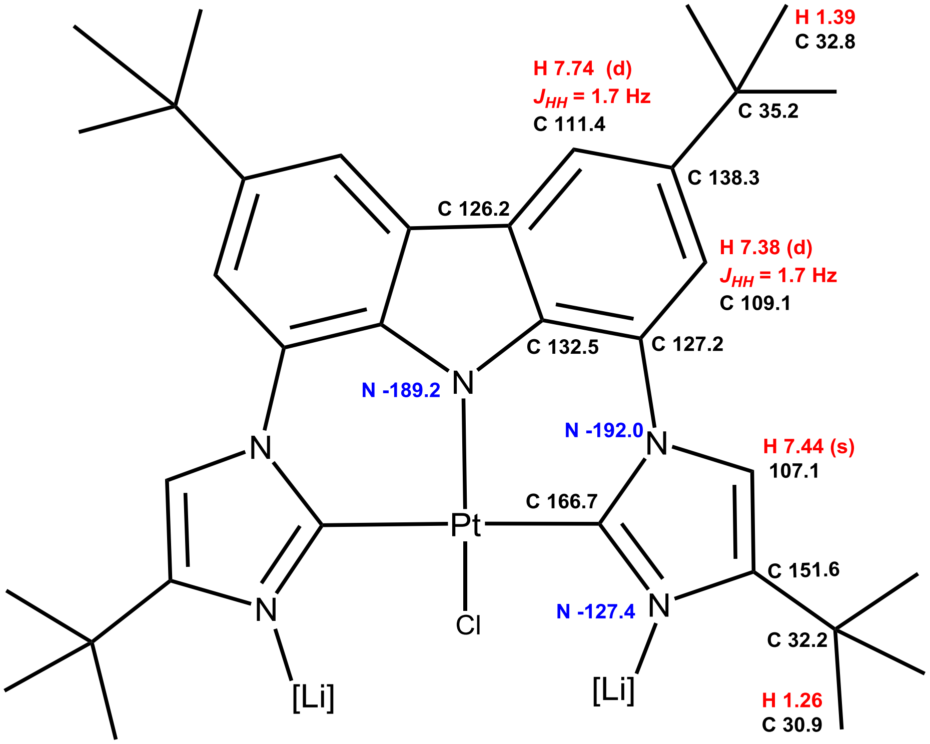

Figure S5: NMR data for **8-Pt** in THF (0.6 mL) and d_6-benzene (0.1 mL).

^{1}H - ^{15}N gHMBCAD[a]		
H	bonds	N
7.74	4	-189.2
7.44	2	-192.0
7.44	3	-127.4
7.38	3	-192.0
[a]J_{nxh}= 5 Hz J_{1xh}= 90 Hz		

^{1}H-^{13}C HSQCAD	
H	C
7.74	111.4
7.44	107.1
7.38	109.1
1.39	32.8
1.26	30.9

gCOSY
7.74↔7.38

^{1}H - ^{13}C gHMBCAD[b]		
H	bonds	C
7.74	3	109.1
7.74	2	126.2
7.74	4	127.2(w)
7.74	3	132.5
7.44	2	151.6
7.44	3	166.7
7.38	3	111.4
7.38	2	127.2
7.38	3	132.5
1.39	1,3	32.8
1.39	2	35.2
1.39	3	138.3
1.26	1,3	30.9
1.26	2	32.2
1.26	3	151.6
[b]J_{nxh} = 8.0 Hz J_{1xh} = 146.0 Hz		

Red = Proton, Black = carbon, Blue = Nitrogen

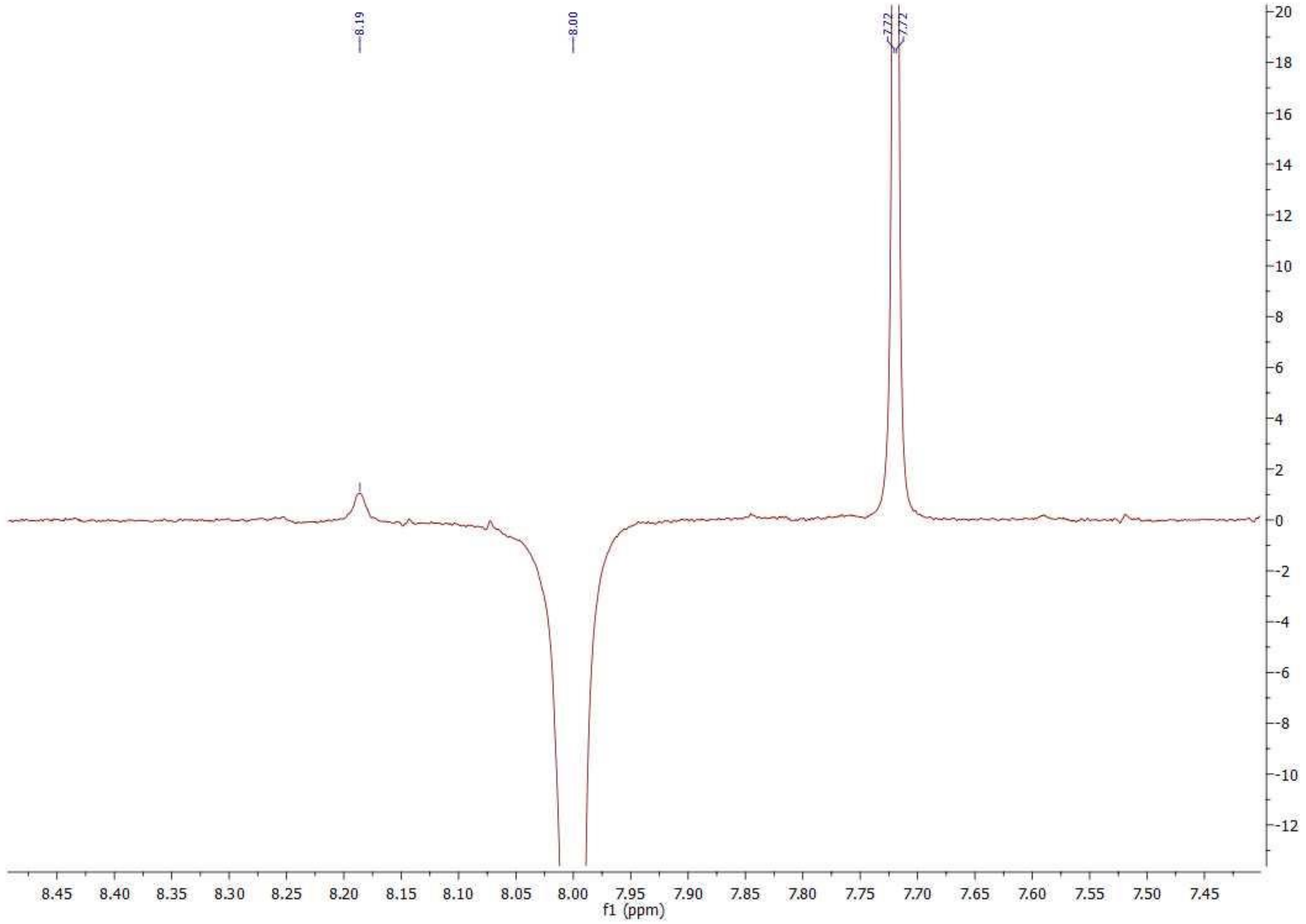

Figure S6: ROESY 1D spectrum of **6-Pt**, irradiating peak at 8.01, C4 on MNHC imidazole and seeing the correlating peaks at 7.72 ppm from the carbazole, and 8.19 ppm from the NH on the PNHC.

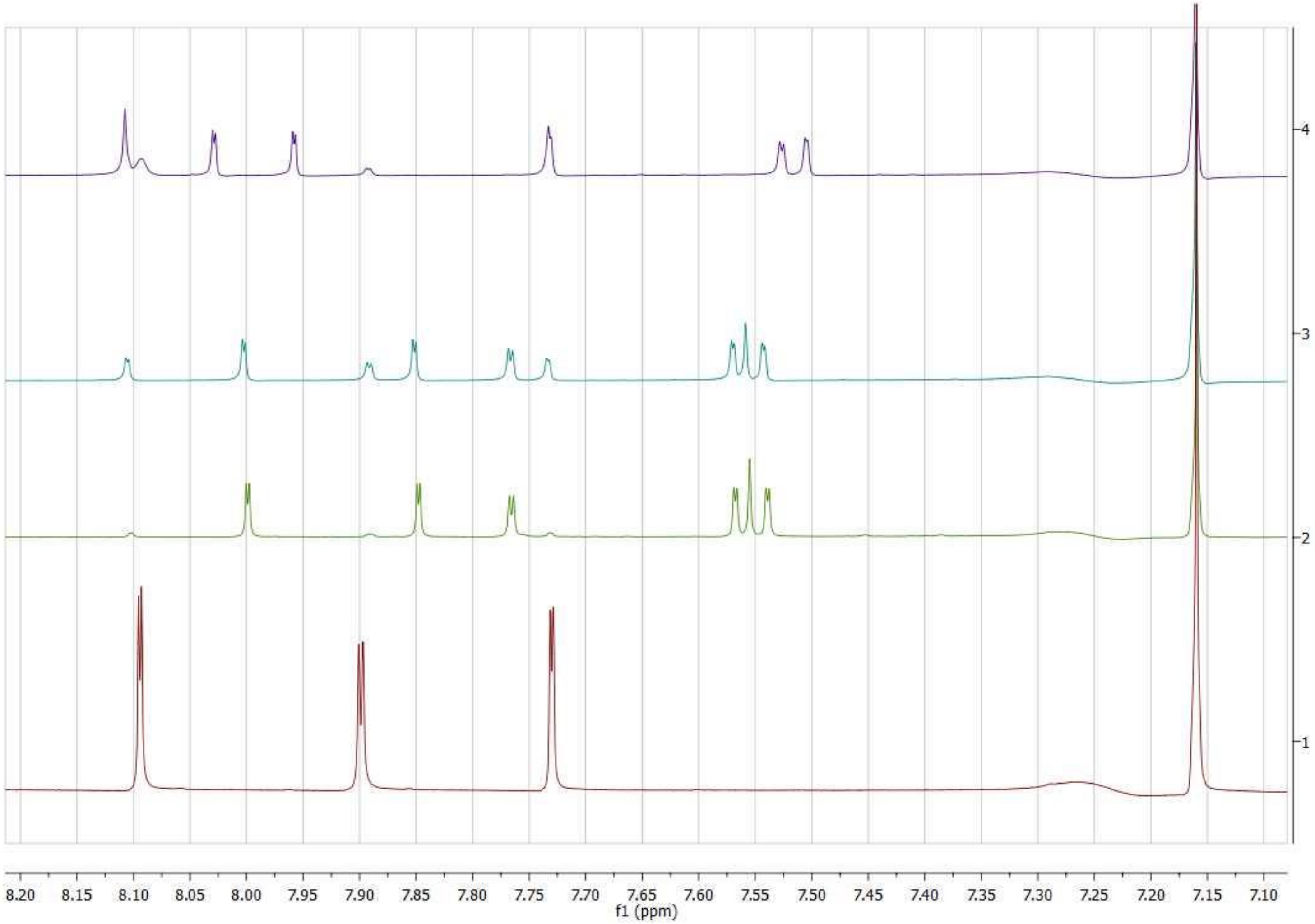

Figure S7: ^{1}H NMR spectra of **4-PtCl** (red, 1), **7-Pt** (green, 2), 19 h after bubbling H_2 in solution (blue, 3) mostly still **7-Pt** with some **4-PtCl**, NMR after addition of AgOTf (1.25 equiv) (purple, 4), formation of dimer **6-Pt**.

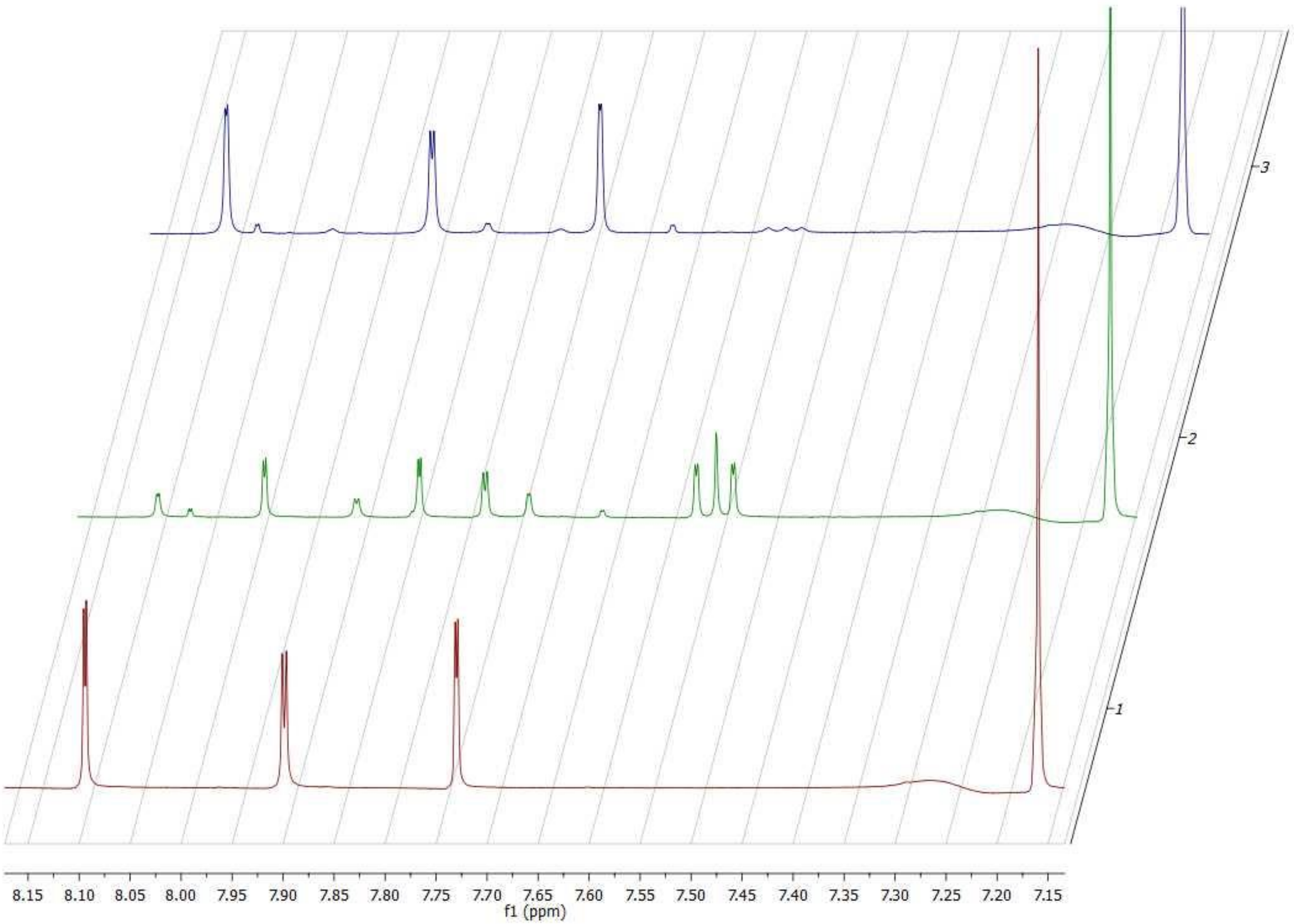

Figure S8: ^{1}H NMR spectra of **4-PtCl** (red) in THF (0.6 mL) and d_6-benzene(0.1 mL), ^{1}H NMR of **7-Pt** (green), formed from **4-PtCl** and *n*-BuLi (2.5 M, 1 equiv), ^{1}H NMR after bubbling ethylene gas through solution for 4 minutes (blue), showing mostly **4-PtCl**.

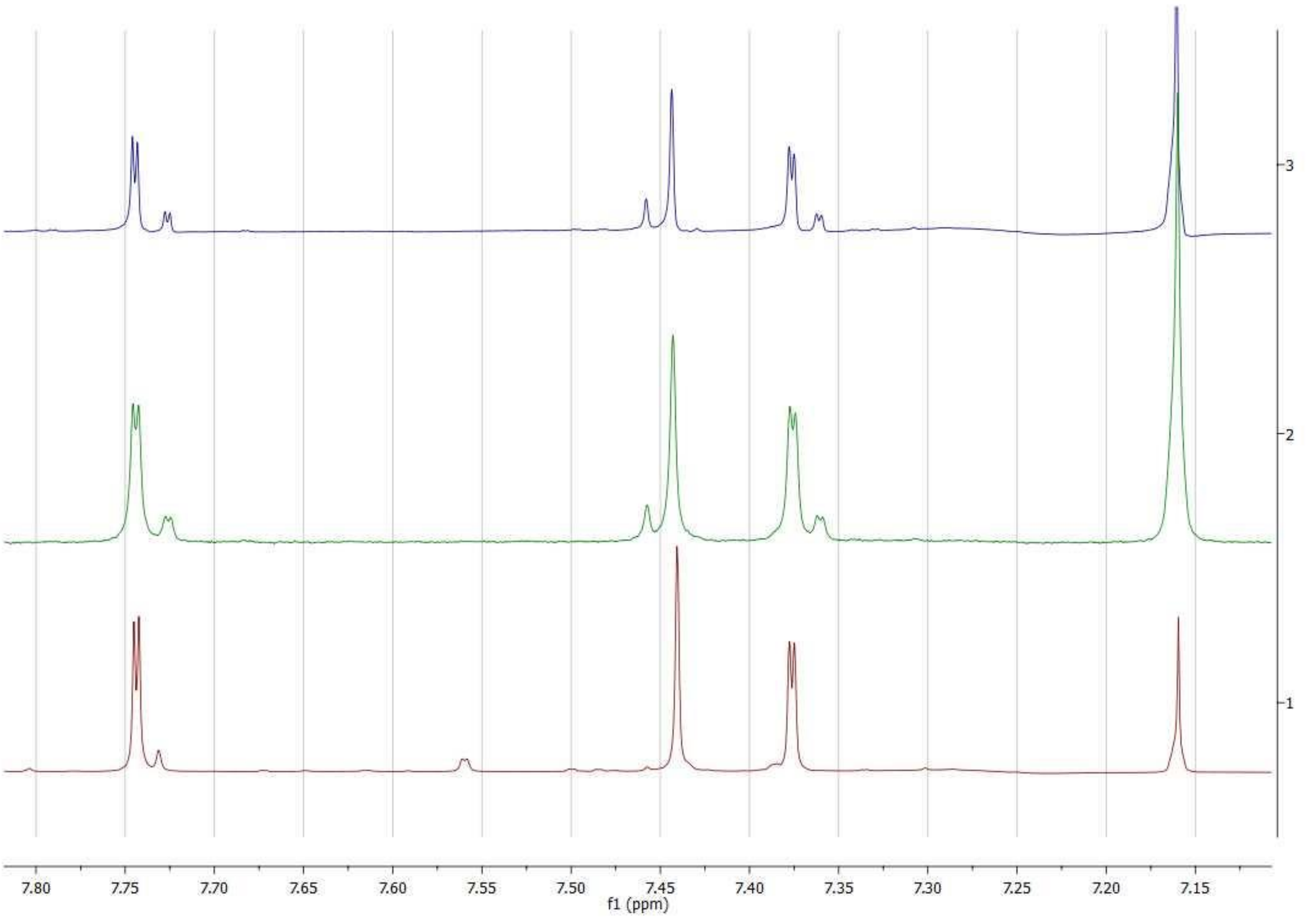

Figure S9: ^{1}H NMR spectra of **8-Pt** (red, 1) in THF (0.6 mL) and d_6-benzene(0.1 mL), after addition of 1-heptene (20 equiv) (green, 2), after heating for 4 h at 70 °C (purple, 3).

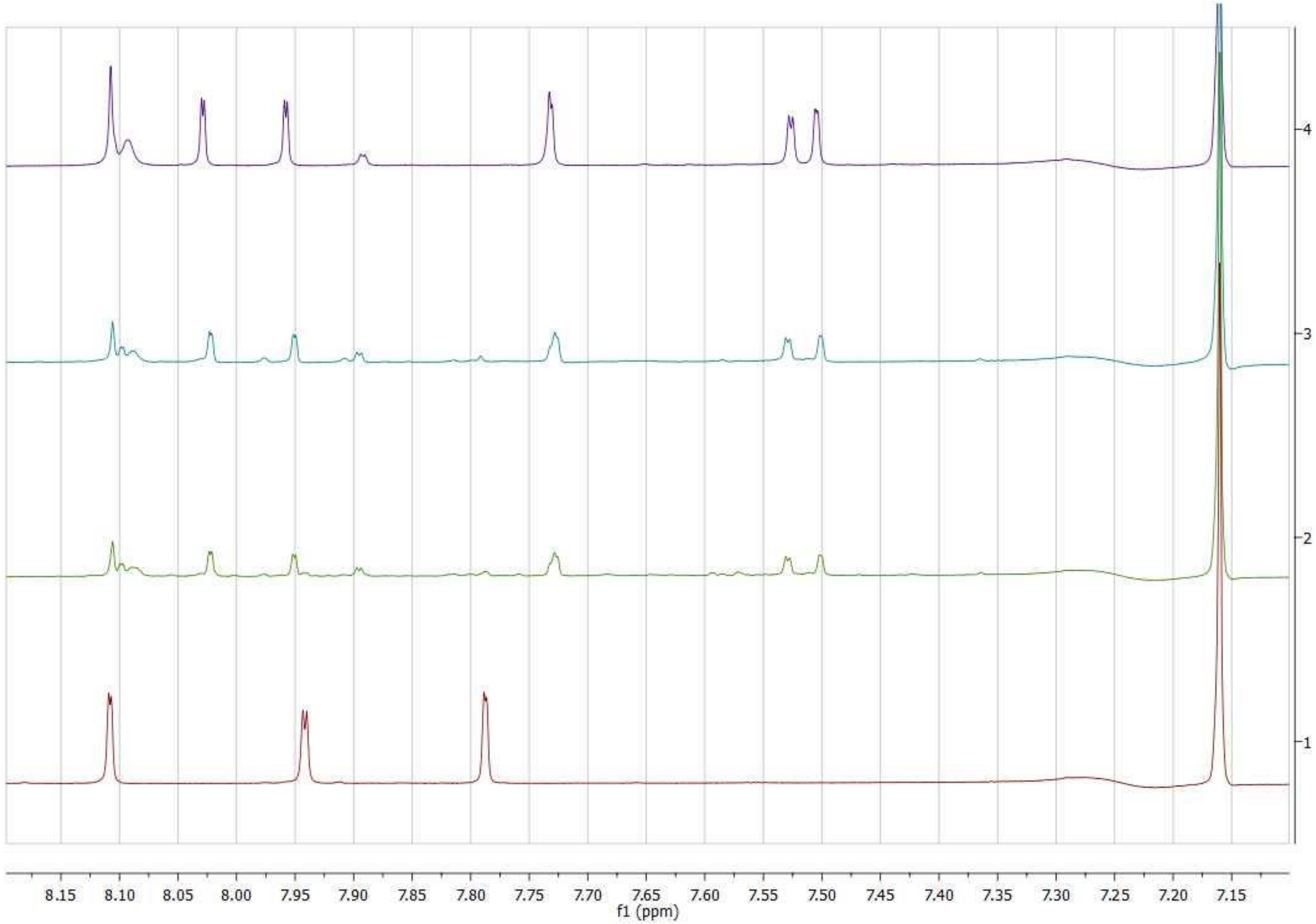

Figure S10: ^{1}H NMR spectra of **4-PtOTf** (red, 1) in THF (0.6 mL) and d_6-benzene (0.1 mL), after addition of LiN(iPr)$_2$ (1 equiv) (green, 2), after sitting at room temperature for 27 h (blue, 3), **6-Pt** (purple, 4) from Figure S7.

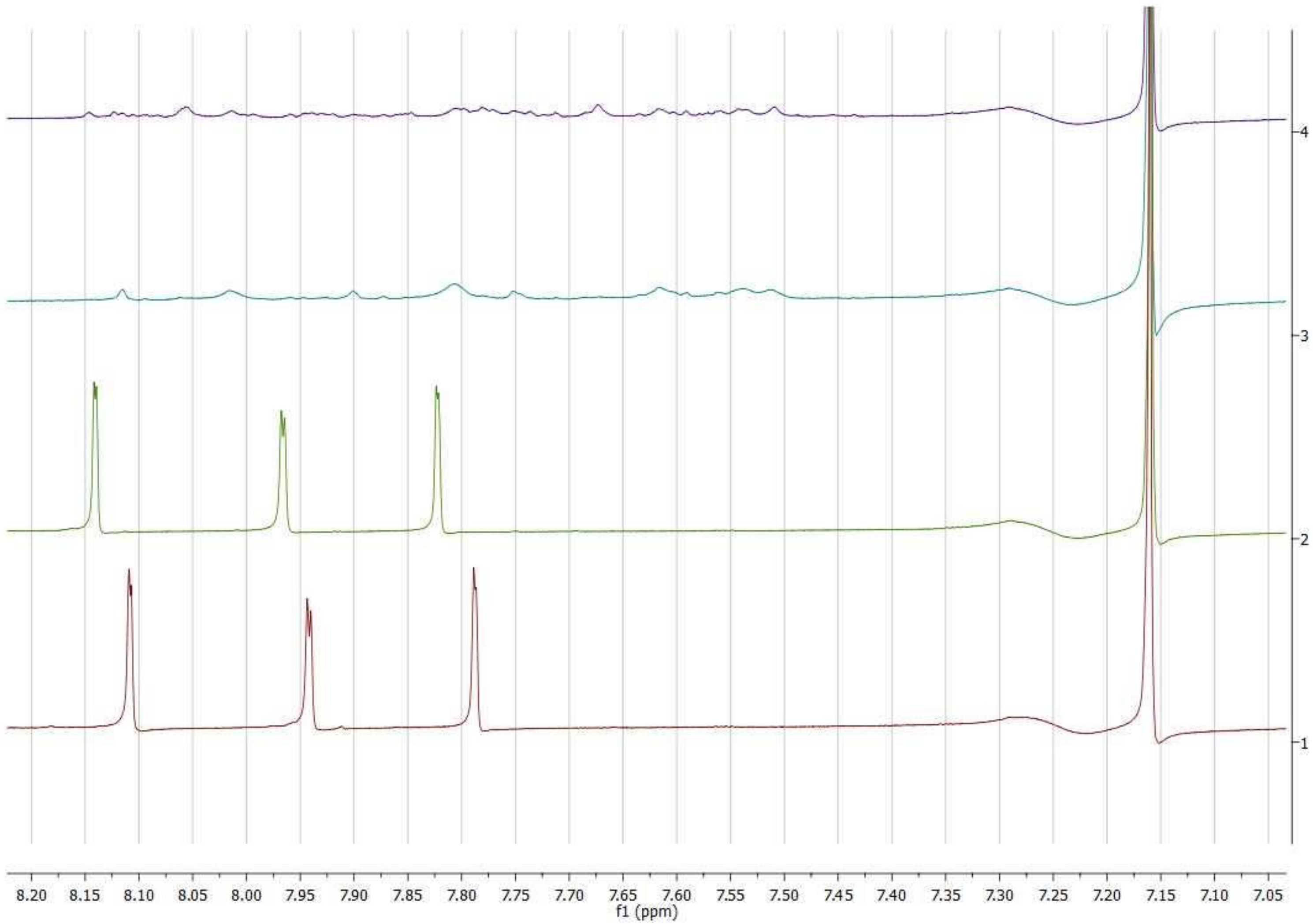

Figure S11: ^{1}H NMR spectra of **4-PtOTf** (red, 1) in THF (0.6 mL) d_6-benzene (0.1 mL); [**4-Pt(CH_3CN)**]$^+$ **OTf**$^-$ (green, 2), after addition of CH_3CN (10 equiv); 5 minutes after addition of LiN(iPr)$_2$ (1 equiv) (blue, 3); 24 hours after addition of LiN(iPr)$_2$ (1 equiv) (purple, 4).

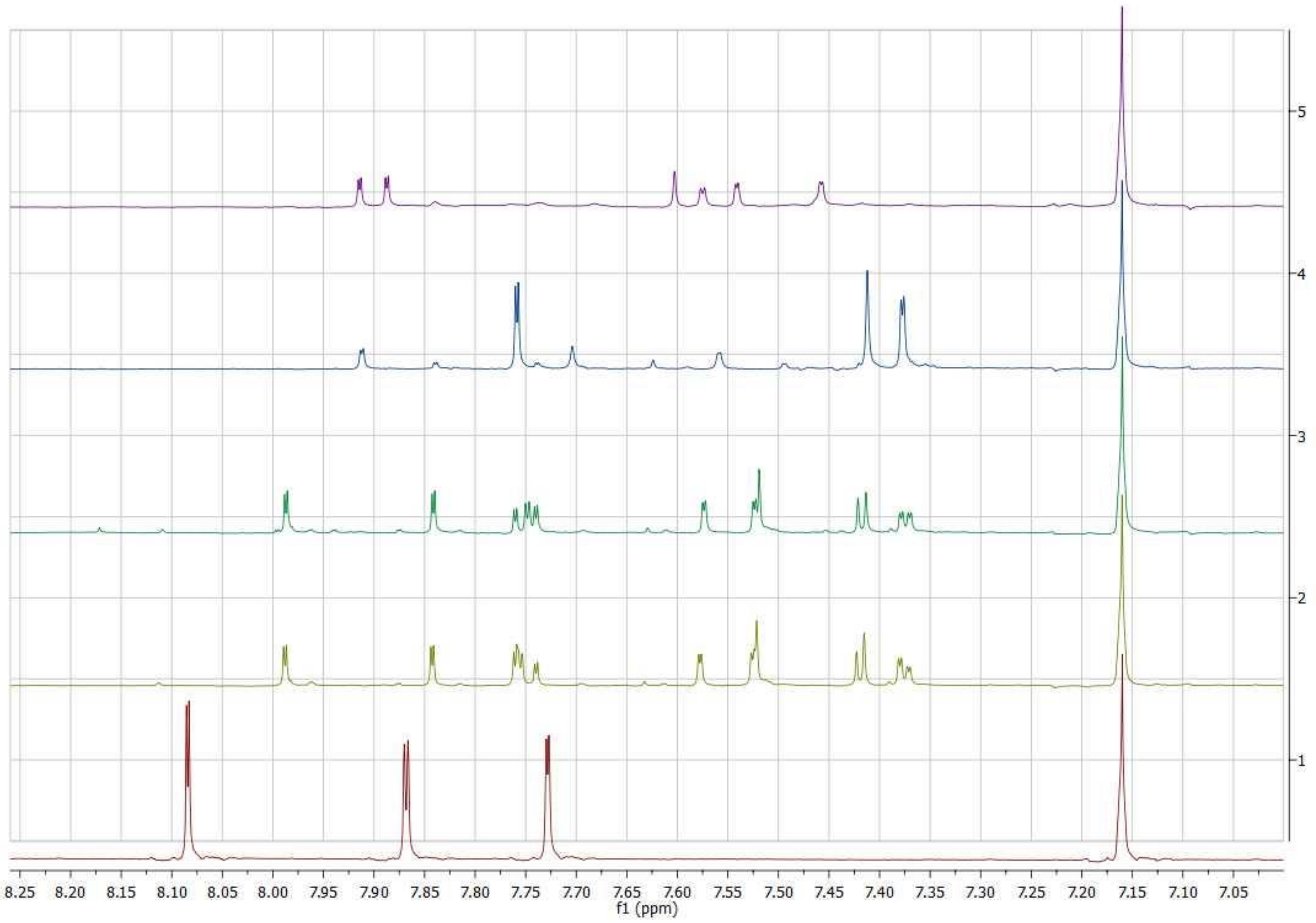

Figure S12: Starting **4-PdCl** complex in THF (0.6 mL) and d_6-benzene (0.1 mL) (red, 1); after addition of $LiN(iPr)_2$ (~1 equiv), giving a mixture of **7-Pd** and **8-Pd** (light green, 2); after addition of 1-heptene and heating at 70 °C for 16 h (green, 3) giving the same mixture of **7-Pd** and **8-Pd**; after addition of more $LiN(iPr)_2$ (1 equiv), giving **8-Pd** with impurities; after addition of AgOTf (1.5 equiv), giving what appears to be deprotonated dimer **6-Pd**.

General experimental

Manipulations were carried out in a nitrogen-filled glovebox or using Schlenk techniques unless otherwise specified. THF and diethyl ether were distilled from sodium and benzophenone, whereas dichloromethane and hexane were distilled from calcium hydride under nitrogen. d_6-benzene and $CDCl_3$ were purchased from Cambridge Isotope Labs, dried over calcium hydride, and vacuum transferred prior to use. d_6-acetone and CD_2Cl_2 were purchased from Cambridge Isotope Labs, was further deoxygenated by bubbling nitrogen gas through the liquid and transferred to a glovebox. ^{1}H NMR spectra were obtained on Varian spectrometers, 600 MHz. ^{13}C NMR and 2D NMR spectra were obtained on the same instrument. Both ^{1}H NMR and ^{13}C NMR chemical shifts were reported in parts per million downfield from tetramethylsilane and referenced to the solvent resonances (^{1}H NMR: 7.16 ppm for $C_6\mathit{H}D_5$ 7.27 ppm for $C\mathit{H}Cl_3$, 5.32 ppm for $C\mathit{H}DCl_2$ and 2.05 ppm for d_5-acetone . ^{13}C NMR: 128.39 ppm for C_6D_6, 77.23 ppm for $\mathit{C}DCl_3$, 54.00 ppm for CD_2Cl_2, and 29.92 ppm for d_6-acetone), where ^{1}H NMR chemical shifts are followed by multiplicity, coupling constants *J* in hertz, and integration in parentheses.

^{15}N chemical shifts on unlabeled materials were determined from gradient HSQC or HMBC experiments run on a Varian INOVA 600 MHz spectrometer. Sweep widths and the number of increments in the ^{15}N dimension were chosen so as to give digital resolutions in the ^{15}N dimension of less than 1.5 ppm. The ^{15}N chemical shift of a standard reference sample of formamide solution in d_6-dimethylsulfoxide (90%) was determined and set to be −267.8 ppm. Then, using the same sweep width and offsets, samples of nitromethane (1.0 M in $CDCl_3$) and quinine (0.5 M in $CDCl_3$) gave ^{15}N chemical shifts of −4.2 for CH_3NO_2 and −72.4 and −349.2 ppm for the sp^2 and sp^3 hybridized nitrogens of quinine, respectively.

Synthesis of **6-Pd**

To a J. Young NMR tube in a glovebox was added **4-PdCl** (40.1 mg, 0.060 mmol), sodium tert-butoxide (7.0 mg, 0.072 mmol), and C_6D_6 (2.0 mL). After 45 minutes the reaction was transferred to a vial in the glovebox and solvent was reduced to approximately 0.75 mL. Et_2O was then allowed to mix by vapor diffusion in a freezer. The yellow crystals were filtered through a pipet loaded with cotton and washed with pentanes (3 × 0.25 mL), then dissolved in Et_2O and transferred to a vial and dried under oil pump vacuum with P_2O_5 yielding **6-Pd** (19.3 mg, 0.015 mmol, 50 % yield). Anal. calcd. for $C_{68}H_{86}N_{10}Pd_2$ (H_2O) (mol. wt. 1274.36): C, 64.09; H, 6.96; N, 10.99. Found C, 63.95; H, 6.91; N, 10.93.

Synthesis of **6-Pt**

To a J. Young NMR tube in a glove box was added **4-PtCl** (50.0 mg, 0.066 mmol), sodium *tert*-butoxide (6.7 mg, 0.070 mmol), and C_6D_6 (2.5 mL). The solution was heated in a 70 °C oil bath for 25 minutes,

which was then transferred into a vial in the glovebox and solvent was reduced to approximately 0.75 mL. Et_2O was allowed to mix by vapor diffusion in a freezer where crystals formed. The yellow crystals were filtered through a pipet loaded with cotton and washed with pentanes (3 × 0.1 mL), then transferred to a vial by dissolving in Et_2O and dried under oil pump vacuum with P_2O_5 yielding **6-Pt** (27.4 mg, 0.019 mmol, 56% yield). Anal. calcd. for $C_{68}H_{86}N_{10}Pt_2$ $(H_2O)_2$ (mol. wt. 1469.70): C, 55.57; H, 6.17; N, 9.53. Found C, 55.43; H, 6.03; N, 9.60.

Table S1: Crystal data and structure refinement for **6-Pd**.

Identification code	**6-Pd**	
Empirical formula	C46.50 H56 Cl N5 Pd	
Formula weight	826.81	
Temperature	100(2) K	
Wavelength	1.54178 Å	
Crystal system	Monoclinic	
Space group	C2/c	
Unit cell dimensions	a = 28.1402(8) Å	α = 90°.
	b = 15.7664(5) Å	β = 94.048(2)°.
	c = 19.0083(6) Å	γ = 90°.
Volume	8412.4(4) $Å^3$	
Z	8	
Density (calculated)	1.306 Mg/m^3	
Absorption coefficient	4.424 mm^{-1}	
F(000)	3464	
Crystal size	0.15 x 0.11 x 0.08 mm^3	
Theta range for data collection	3.15 to 64.88°.	
Index ranges	-28<=h<=32, -18<=k<=18, -21<=l<=22	
Reflections collected	25380	
Independent reflections	6990 [R(int) = 0.0355]	
Completeness to theta = 64.88°	97.9 %	
Absorption correction	Multi-scan	
Max. and min. transmission	0.7185 and 0.5566	
Refinement method	Full-matrix least-squares on F^2	
Data / restraints / parameters	6990 / 37 / 502	
Goodness-of-fit on F^2	1.052	
Final R indices [I>2sigma(I)]	R1 = 0.0473, wR2 = 0.1300	
R indices (all data)	R1 = 0.0576, wR2 = 0.1394	
Largest diff. peak and hole	1.071 and -1.478 e.$Å^{-3}$	

Table S2: Crystal data and structure refinement for **6-Pt**.

Identification code	**6-Pt**	
Empirical formula	C43 H56 N5 Pt	
Formula weight	838.02	
Temperature	100(2) K	
Wavelength	0.71073 Å	
Crystal system	Monoclinic	
Space group	C2/c	
Unit cell dimensions	a = 28.6827(19) Å	α = 90°.
	b = 15.2306(10) Å	β = 93.9130(10)°.
	c = 19.5645(13) Å	γ = 90°.
Volume	8526.9(10) $Å^3$	
Z	8	
Density (calculated)	1.306 Mg/m^3	
Absorption coefficient	3.324 mm^{-1}	
F(000)	3416	
Crystal size	0.34 x 0.13 x 0.11 mm^3	
Crystal color, habit	Orange Block	
Theta range for data collection	1.87 to 27.49°.	
Index ranges	-37<=h<=36, -19<=k<=19, -19<=l<=25	
Reflections collected	48497	
Independent reflections	9509 [R(int) = 0.0411]	
Completeness to theta = 25.00°	99.6 %	
Absorption correction	Multi-scan	
Max. and min. transmission	0.7113 and 0.3978	
Refinement method	Full-matrix least-squares on F^2	
Data / restraints / parameters	9509 / 0 / 526	
Goodness-of-fit on F^2	1.048	
Final R indices [I>2sigma(I)]	R1 = 0.0283, wR2 = 0.0675	
R indices (all data)	R1 = 0.0403, wR2 = 0.0752	
Largest diff. peak and hole	1.246 and -0.696 e.$Å^{-3}$	
